# Briarane Diterpenoids Isolated from Octocorals between 2014 and 2016

**DOI:** 10.3390/md15020044

**Published:** 2017-02-17

**Authors:** Yin-Di Su, Jui-Hsin Su, Tsong-Long Hwang, Zhi-Hong Wen, Jyh-Horng Sheu, Yang-Chang Wu, Ping-Jyun Sung

**Affiliations:** 1 National Museum of Marine Biology and Aquarium, Pingtung 944, Taiwan; gobetter04@gmail.com (Y.-D.S.); x2219@nmmba.gov.tw (J.-H.S.); 2 Graduate Institute of Marine Biology, National Dong Hwa University, Pingtung 944, Taiwan; 3 Graduate Institute of Natural Products, College of Medicine, Chinese Herbal Medicine Research Team, Healthy Aging Research Center, Chang Gung University; Research Center for Chinese Herbal Medicine, Research Center for Food and Cosmetic Safety, and Graduate Institute of Health Industry Technology, College of Human Ecology, Chang Gung University of Science and Technology; Department of Anesthesiology, Chang Gung Memorial Hospital, Taoyuan 333, Taiwan; htl@mail.cgu.edu.tw; 4 Department of Marine Biotechnology and Resources, National Sun Yat-sen University, Kaohsiung 804, Taiwan; wzh@mail.nsysu.edu.tw; 5 Graduate Institute of Natural Products, Kaohsiung Medical University, Kaohsiung 807, Taiwan; 6 Research Center for Natural Products and Drug Development, Kaohsiung Medical University, Kaohsiung 807, Taiwan; 7 Department of Medical Research, Kaohsiung Medical University Hospital, Kaohsiung 807, Taiwan; 8 Chinese Medicine Research and Development Center, China Medical University Hospital, Taichung 404, Taiwan

**Keywords:** briarane, octocoral, *Briareum*, *Dichotella*, *Ellisella*, *Junceella*, *Pennatula*

## Abstract

The structures, names, bioactivities, and references of 124 briarane-type natural products, including 66 new metabolites, isolated between 2014 and 2016 are summarized in this review article. All of the briarane diterpenoids mentioned in this review were isolated from octocorals, mainly from *Briareum violacea*, *Dichotella gemmacea*, *Ellisella dollfusi*, *Junceella fragilis*, *Junceella gemmacea*, and *Pennatula aculeata*. Some of these compounds exhibited potential biomedical activities, including anti-inflammatory activity, antibacterial activity, and cytotoxicity towards cancer cells.

## 1. Introduction

**Figure 1:**
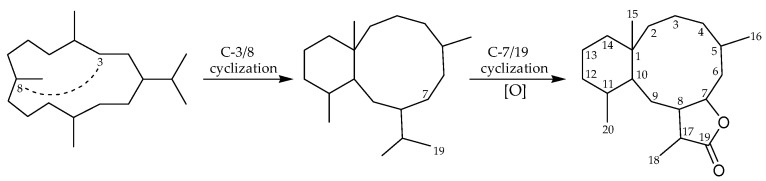
Possible biogenetic origin of briarane-type metabolites. The numbering system shown is that presently in use [1].

Following previous review articles from our research group focused on marine-origin briarane-type natural products [1,2,3,4,5], this review covers the literature from 2014 to January 2017, and describes 124 briarane-related diterpenoids (including 66 new metabolites), most of which are characterized by the presence of a γ-lactone moiety fused to a bicyclo[8.4.0] ring system, obtained from various octocorals (Figure 1), mainly *Briareum violacea*, *Briareum* spp. *Dichotella gemmacea*, *Ellisella dollfusi*, *Junceella fragilis*, *Junceella gemmacea*, and *Pennatula aculeata*. Many of these compounds exhibited interesting bioactivities in vitro, which might indicate a potential for use in biomedical applications. This survey of briarane-related compounds is presented taxonomically according to genus and species.

## 2. Alcyonacea

### 2.1. Briareum violacea (Family Briareidae)

The taxonomic position of octocorals affiliated with the genus *Briareum* (=*Asbestia*, *Pachyclavularia*, and *Solenopodium*) [6] has been found to be situated near the transition between Alcyonacea and Gorgonacea, in both taxonomic and chemical terms [6,7,8]. In 1977, briarein A, the first briarane-type diterpenoid identified, was isolated from the Caribbean octocoral *Briareum asbestinum* [9], and since then *Briareum* has been the main organism from which briarane-type natural products have been obtained.

**Figure 2:**
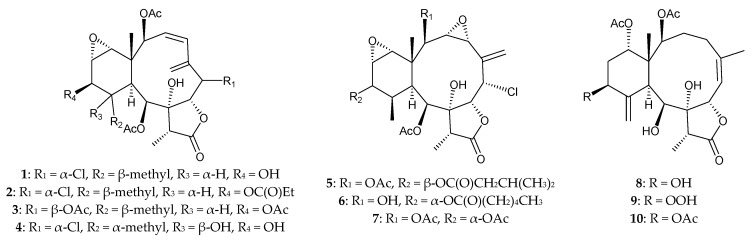
Structures of briaviolides A–J (**1**–**10**).

Sixteen briarane diterpenoids, including 10 new 8-hydroxybriaranes, briaviolides A–J (**1**–**10**) (Figure 2), and six known metabolites, solenolides A [10] and D (=briaexcavatolide E) [1,10,11,12], excavatolide A [13], briaexcavatolide I [12], 4β-acetoxy-9-deacetylstylatulide lactone, and 9-deacetylstylatulide lactone [14], were isolated from the octocoral *Briareum violacea*, collected from the waters of Taiwan [15]. The structures of new briaranes **1**–**10** were established by chemical and spectroscopic methods, and determination of the absolute configuration of briaviolide A (**1**) was completed by X-ray diffraction analysis of its monobenzoyl derivative [15]. At a concentration of 10 μg/mL, briaranes **5** and **9** were found to exert moderate inhibitory activities on elastase release (inhibition rate = 26.0% and 28.8%, respectively) and superoxide anion production (inhibition rate = 34.2% and 28.7%, respectively) by human neutrophils [15].

### 2.2. Briareum *sp.*

**Figure 3:**
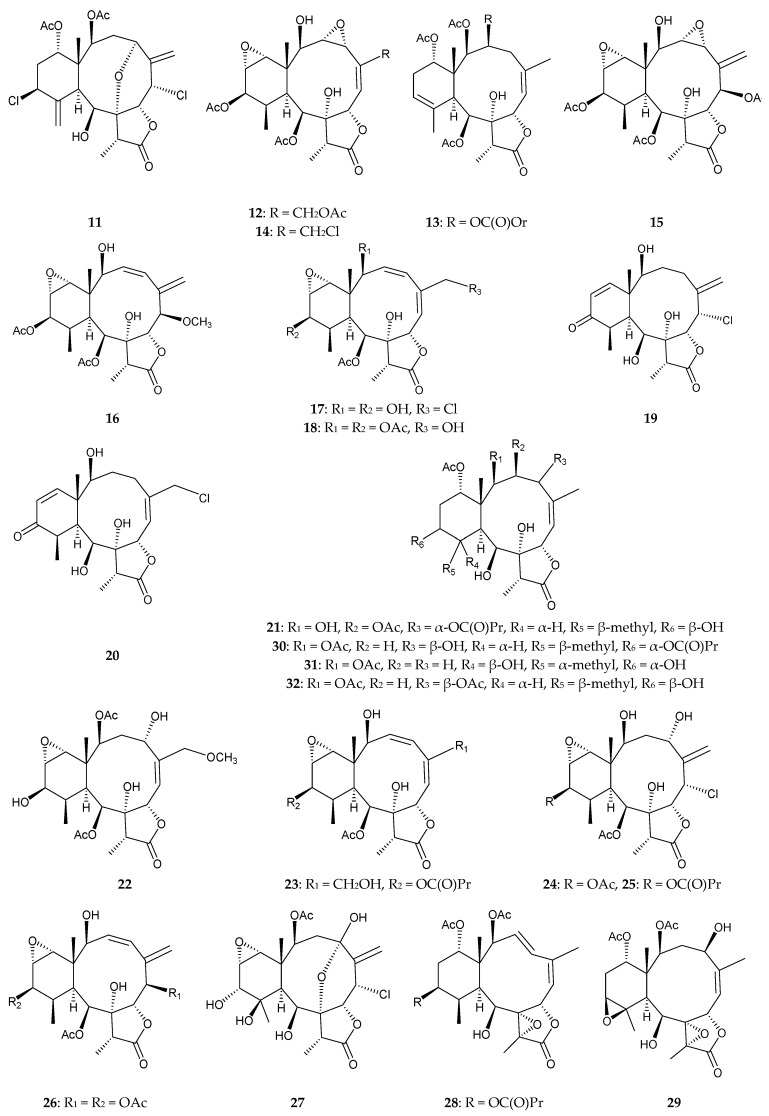
Structures of briarenolides J–Y (**11**–**26**) and ZI–ZVI (**27**–**32**).

In continuing chemical studies of the constituents of an octocoral identified as *Briareum* sp. collected from the southern waters of Taiwan, 22 new briarane derivatives, briarenolides J–Y (**11**–**26**) and ZI–ZVI (**27**–**32**), were obtained, and their structures determined based on analysis of their spectroscopic data (Figure 3) [16,17,18,19,20]. Briarenolide J (**11**) was the first 12-chlorinated diterpenoid to be isolated from *Briareum* sp. [16]. The relationships between the ^1^H and ^13^C NMR chemical shifts of 2-hydroxybriaranes possessing a Δ^3,5(16)^-conjugated diene moiety or a Δ^3,5^-conjugated moiety have been summarized [18]. Briarane **11** has been shown to inhibit superoxide anion generation and elastase release, with IC_50_ values of 15.0 and 10.0 μM, respectively [16]. In macrophage cells, briaranes **12**–**14**, **17**, **20**–**24**, **26**, **28** and **32** were found to reduce the level of iNOS to 23.7%, 31.7%, 49.6%, 58.4%, 57.4%, 53.5%, 41.9%, 47.3%, 50.1%, 54.3%, 47.2% and 55.7%, respectively, at a concentration of 10 μM [17,18,19,20]. Briaranes **15**, **17**, **21**–**24**, and **26** were found to reduce the level of COX-2 to 53.9%, 59.1%, 59.3%, 26.1%, 35.6%, 58.1% and 55.4%, respectively, at a concentration of 10 μM [18,19].

## 3. Gorgonacea

### 3.1. Dichotella gemmacea (Family Ellisellidae)

**Figure 4:**
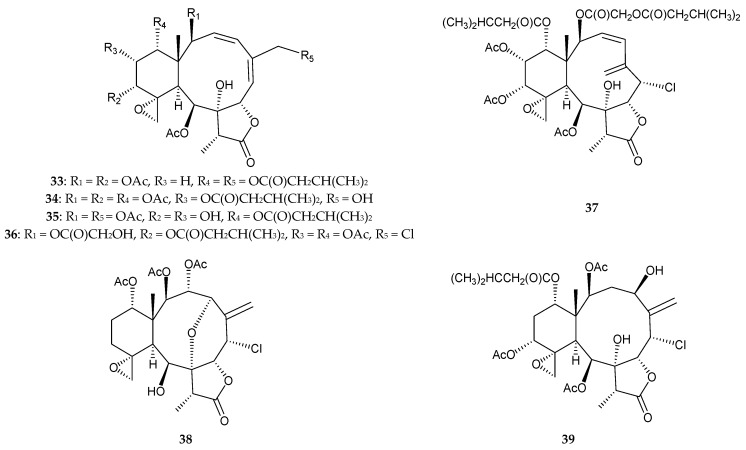
Structures of gemmacolides AS–AY (**33–39**).

In 2014, Zhang et al. reported the isolation of seven new briarane derivatives, which were named gemmacolides AS–AY (**33–39**) (Figure 4), along with 10 known analogues, gemmacolides L [21], X (=dichotellide T) [22,23], AH, AJ, AO, AQ [24], junceellolides C and D [25], junceellin (=junceellin A) [25,26,27,28,29,30,31,32], and frajunolide K [33], from the South China Sea gorgonian coral, *D. gemmacea* [34]. Structural determination of new briaranes **33–39** was conducted using spectroscopic methods, and their absolute configurations were established based on the results of electronic circular dichroism (ECD) experiments [34]. Briarane **37** was found to exert a cytotoxic effect towards MG-63 (human osteosarcoma) cells, with an IC_50_ value of 7.2 μM [34].

**Figure 5:**
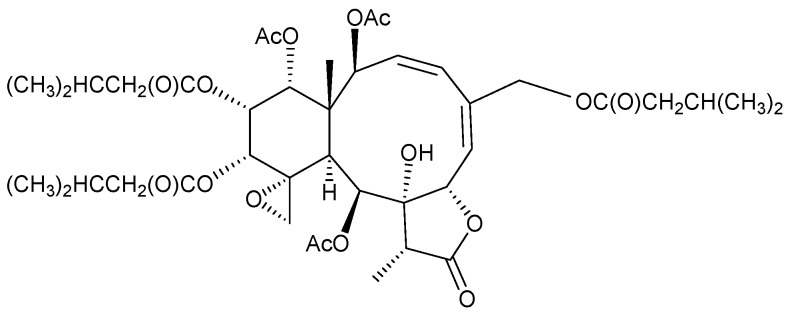
Structure of dichotellide V (**40**).

A new briarane, dichotellide V (**40**) (Figure 5), along with four known briarane analogues, gemmacolide N [35], dichotellide J [23], junceellin A (=junceellin) [25,26,27,28,29,30,31,32], and junceellolide A [25], were isolated from *Dichotella gemmacea*, collected from Meishan Island, Hainan Province, China [36]. The structure of new briarane 40 was determined by spectroscopic methods, and none of the above compounds exhibited a cytotoxic effect on A549 (human epithelial lung carcinoma), BGC823 (human gastric cancer), H1975 (human non-small cell lung cancer), HeLa (human cervix adenocarcinoma), MCF7 (human mammary gland adenocarcinoma), or U-937 (human histiocytic lymphoma) tumor cells [36].

Eight known briaranes, junceellolide D [25], (+)-11β,20β-epoxyjunceellolide D, (−)-11β,20β- epoxy-4-deacetoxyjunceellolide D [30,37], junceol A [38], juncins H and K [39,40], praelolide [25,29,30,31,32,41,42], and junceellin (=junceellin A) [25,26,27,28,29,30,31,32], were obtained from *D. gemmacea*, collected from Meishan Island, Hainan Province, China in April 2009 [43]. Junceellolide D and praelolide showed antifouling activity against the settlement of larvae of barnacle *Balanus amphitrite* with EC_50_ values of 14.5 and 16.7 μM, respectively. Junceellolide D, (−)-11β,20β-epoxy-4-deacetoxyjunceellolide D, juncin H, and praelolide exhibited lethality towards brine shrimp *Artemia salina* with lethal ratios of 90%, 85%, 60% and 75% at a concentration of 50 μg/mL [43].

**Figure 6:**
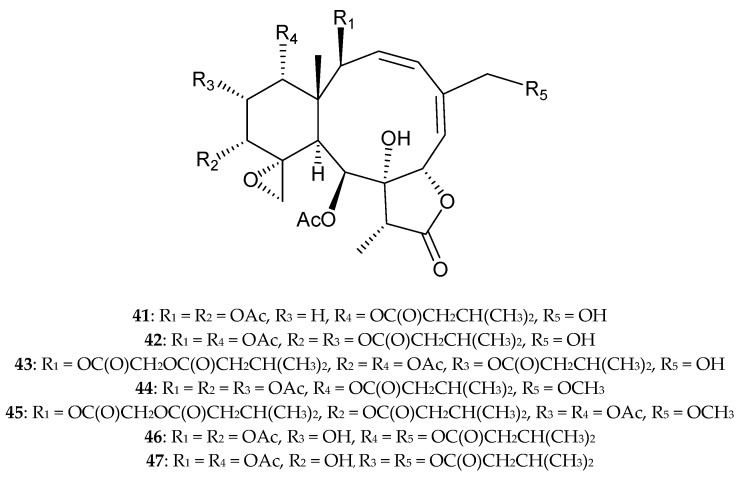
Gemmacolides AZ–BF (**41**–**47**).

In addition, seven new briaranes, gemmacolides AZ–BF (**41**–**47**) (Figure 6), and eight known analogues, dichotellides M and O [23], gemmacolide C [44], juncins P [45] and ZI [46], junceellolides D [25] and K [37], and (−)-4-deacetyljunceellolide D [30], were obtained from *D. gemmacea*, collected from the South China Sea [47]. The structures of new briaranes 41–47 were determined by spectroscopic methods. Briaranes **41**–**44**, **46**, **47**, and dichotellide O, showed cytotoxicity towards A549 cells, with IC_50_ values of 28.3, 24.7, 34.1, 26.8, 25.8, 13.7 and 25.5 μM, respectively. Briaranes **42**, **44**, **46**, **47**, and dichotellide O, showed cytotoxicity towards MG-63 cells, with IC_50_ values of 15.8, 11.4, 30.6, 34.8 and 36.8 μM, respectively. Briarane **44** and dichotellide O exhibited antibacterial activity against the Gram-negative bacterium *Escherichia coli*, while dichotellide O demonstrated actitity against the Gram-positive bacterium *Bacillus megaterium* [47].

### 3.2. Ellisella dollfusi (Family Ellisellidae)

**Figure 7:**
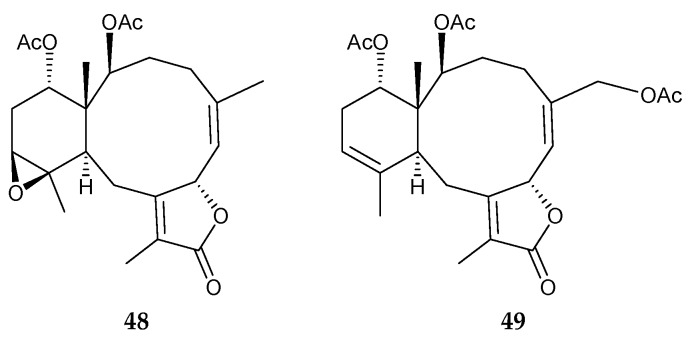
Structures of dollfusilins A (**48**) and B (**49**).

Zhou and coworkers isolated seven briaranes, including two new compounds, dollfusilins A (**48**) and B (**49**) (Figure 7), along with five known analogues, brianthein W [14,48,49], funicolide E [49], 9-deacetylbriareolide H [14,50,51], 9-deacetylstylatulide lactone [14], and umbraculolide A [29,52], from the organic extract of gorgonian coral *Ellisella dollfusi*, collected from the Xisha Sea area of the South China Sea [53]. The structures of new briaranes **48** and **49** were determined through comprehensive analysis of spectroscopic data. Brianthein W exhibited an effect of delayed hatching and notochord growth malformation toxicity towards zebrafish *Danio rerio* embryos with IC_50_ values of 30.6 and 18.9 μg/mL in 48 h, respectively. Funicolide E displayed egg coagulation and delayed hatching toxicity towards zebrafish embryos, with EC_50_ values of 33.6 μg/mL (24 h) and 29.8 μg/mL, respectively [53].

### 3.3. Junceella fragilis (Family Ellisellidae)

**Figure 8:**
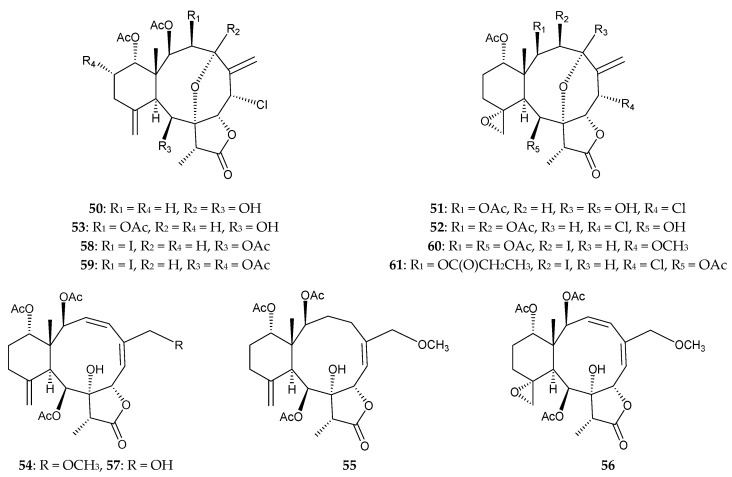
Structures of fragilisinins A–L (**50–61**).

Gorgonian corals belonging to the genus *Junceella* have also been found to be major sources of briarane-related natural diterpenoids [54,55]. The gorgonian *J. fragilis*, collected from the South China Sea, was found to contain 12 new briaranes, fragilisinins A–L (**50**–**61**) [56] (Figure 8), along with seven known analogues, (+)-junceellolide A [30], junceellolide B [25], junceol A [38], junceellonoid D [57,58], fragilide C [59], and frajunolides A [60] and E [33]. The structures of new briaranes **50**–**61** were determined by spectroscopic methods. Briaranes **58**–**61** were the first iodine-containing briarane derivatives to be isolated. The absolute configuration of briarane **50** was confirmed by single-crystal X-ray diffraction data [56]. Briaranes **54**, **55**, **59**, (+)-junceellolide A, and junceellonoid D showed potent antifouling activities against the settlement of barnacle *Balanus amphitrite* larvae, with EC_50_ values of 14.0, 12.6, 11.9, 5.6, and 10.0 μM (LC_50_/EC_50_ = >13, >14.5, >11.5, >33.3, >20), respectively [56].

### 3.4. Junceella gemmacea (Family Ellisellidae)

**Figure 9:**
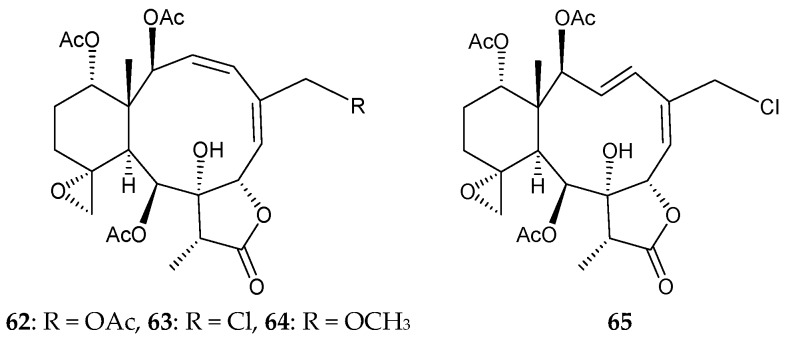
Structures of junceellolides M–P (**62–65**).

Four new briaranes, junceellolides M–P (**62**–**65**) (Figure 9) [61], along with seven known briaranes, junceellolides A–D [25], junceellin A [25,26,27,28,29,30,31,32], praelolide [25,29,30,31,32,41,42], and juncin ZI [46], were isolated from the gorgonian *J. gemmacea*, collected from the South China Sea [61]. The structures, including the absolute configurations, of new briaranes **62**–**65**, were deduced on the basis of spectroscopic analyses, particularly electronic circular dichroism (ECD) experiments, and from biogenetic correlations among briaranes **62**–**65**.

### 3.5. Junceella *sp*. (Family Ellisellidae)

Three known briaranes, junceellin (=junceellin A) [25,26,27,28,29,30,31,32], praelolide [25,29,30,31,32,41,42], and junceellolide A [25], were claimed to have been obtained from a gorgonian coral *Junceella* sp., collected off the Vietnam Thu Island in May 2010 [62]. In the antimicrobial activity test, junceellin and praelolide exhibited weak antibacterial activity against the bacterium *Vibrio parahaemolyticus*. Junceellolide A was also found to display weak antibacterial activity against the bacterium *Candida albicans* [62].

## 4. Pennatulacea

### Pennatula aculeata (Family Pennatulidae)

**Figure 10:**
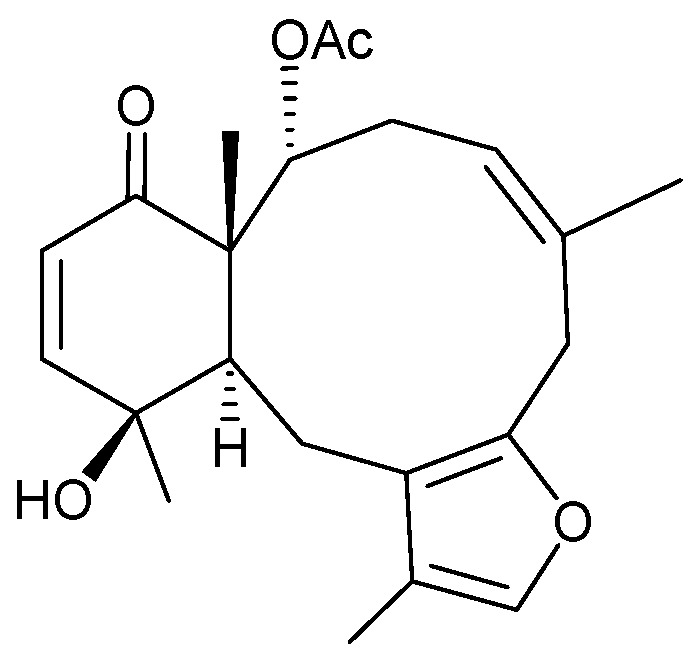
Structure of 2-acetoxyverecynarmin C (**66**).

Investigation of the chemical constituents of *P. aculeata*, collected from Dinawan Island in Sabah, Malaysia, afforded novel briarane 2-acetoxyverecynarmin C (**66**) [63] (Figure 10). The structure of the new briarane **66** was elucidated by analysis of spectroscopic data, and this compound showed moderate inhibitory activity towards COX-1 and COX-2, with IC_50_ values of 44.3 and 47.3 μM, respectively. The 2-acetoxy group in 66 was found to be located on the α-face, relative to Me-15 and H-10, which is a rare occurrence in briarane-related analogues. 

## 5. Conclusions

Since briarein A, the first briarane-type natural product, was prepared from the Caribbean octocoral *Briareum asbestinum* in 1977 [9], over 600 briarane-type diterpenoids have been isolated from a wide variety of marine life to date. A large portion of these natural compounds has been prepared from soft corals belonging to the orders Alcyonacea and Gorgonacea. Compounds of this type of diterpenoid have been demonstrated to possess various bioactivities in vitro, such as anti-inflammatory activity, antibacterial activity, and cytotoxicity towards cancer cells. For example, one of the compounds of this type, excavatolide B [13], has been proven to possess extensive biomedical bioactivities, such as anti-inflammatory, analgesic, the attenuation of rheumatoid arthritis activities, anticancer, and the modulation of the electrophysiological characteristics and calcium homeostasis in atrial myocytes [64,65,66,67]. Due to the structural diversity and biomedical bioactivities, there has been little synthetic work on briarane analogues [68,69]. It is interesting to note that all briaranes reported as having been isolated between 2014 and 2016 were all collected from octocorals distributed in the Indo-Pacific Ocean, particularly from the South China Sea.
